# Supplementary material for: Type 2 Diabetes Risk Perception and Health Behaviors Among Women with History of Gestational Diabetes Mellitus: A Retrospective Analysis
Source: Nutrients. 2025 Oct 25;17(21):3360. doi: 10.3390/nu17213360 (PMC12608729; doi:10.3390/nu17213360)
Supplement: Supplementary file 1 [file nutrients-17-03360-s001.zip › nutrients-3838096-supplementary.pdf]

Table S1. Participant characteristics stratified by perceived risk.

Participant Characteristics	Slight Perceived Risk (n = 77)	Moderate Perceived Risk (n = 108)	High Perceived Risk (n = 57)
Age (SD)	41.4 years (5.6)	41.6 years (5.6)	38.6 years (6.6)
HbA1c (SD)	5.8% (0.1)	5.9% (0.2)	5.9% (0.2)
BMI (SD)	31.5 kg/m2 (6.6)	33.1 kg/m2 (7.3)	33.7 kg/m2 (6.2)
Trial arm			
Control group	36 (47%)	54 (50%)	30 (53%)
SDM group	41 (53%)	54 (50%)	27 (47%)
Race and ethnicity			
AAPI	22 (29%)	32 (30%)	14 (25%)
Black	n <11	n <11	n <11
Hispanic/Latino	18 (23%)	20 (19%)	16 (28%)
Multiracial/Other	n <11	n <11	n <11
White	31 (40%)	48 (44%)	22 (39%)
Family history of diabetes			
No	46 (60%)	54 (50%)	20 (35%)
Yes	31 (40%)	54 (50%)	37 (65%)
Annual household income			
<$100,000	29 (38%)	29 (27%)	22 (39%)
$100,000-$199,999	22 (29%)	39 (36%)	18 (32%)
≥$200,000	22 (29%)	31 (29%)	16 (28%)
Don't Know/Decline to answer	n <11	n <11	n <11
Personal risk perception of T2D	N=77	N=108	N=57
Slight chance	77 (100%)	0 (0%)	0 (0%)
Moderate chance	0 (0%)	108 (100%)	0 (0%)
High chance	0 (0%)	0 (0%)	57 (100%)
Physical activity	N=77	N=108	N=56
No	12 (16%)	20 (19%)	n <11
Yes	65 (84%)	88 (82%)	46 (82%)
Sugar-sweetened beverage consumption	N=77	N=105	N=56
None	26 (34%)	41 (39%)	19 (34%)
<1/day	40 (52%)	47 (45%)	30 (54%)
≥1/day	11 (14%)	17 (16%)	n <11
Ultra-processed food consumption	N=77	N=108	N=56
Rarely/never	32 (42%)	32 (30%)	18 (32%)
Sometimes	38 (49%)	58 (54%)	22 (39%)
Often	n <11	18 (17%)	16 (29%)
Consumption of meals prepared outside the home in last 7 days	N=77	N=108	N=57
median (IQR)	3 meals (1-4)	3 meals (2-5)	3 meals (2-5)
